# Suboccipital neuralgia after C1 pedicle screw insertion: do we neglect atlantooccipital joint violation? - Case report and literature review

**DOI:** 10.1186/s12893-019-0551-3

**Published:** 2019-07-23

**Authors:** Jun-Song Yang, Jing Li, Hao Chen, Peng Liu, Chu Chen, Tuan-Jiang Liu, Lei Chu, Ding-Jun Hao

**Affiliations:** 1 Department of Spine Surgery, Honghui Hospital, Xi’an Jiaotong University, No. 76 Nanguo Road, Xi’an, 710054 Shaanxi China; 2 Department of Anesthesiology, Honghui Hospital, Xi’an Jiaotong University, No. 76 Nanguo Road, Xi’an, 710054 Shaanxi China; 3 Department of Clinical Laboratory, Honghui Hospital, Xi’an Jiaotong University, No. 76 Nanguo Road, Xi’an, 710054 Shaanxi China; 4 Department of orthopaedics, the second affiliated Hospital of Chongqing Medical University, No. 76 Linjiang Road, Chongqing, 400010 China

**Keywords:** Occipital neuralgia, Complications, Atlantoaxial instability, Atlantoaxial fixation, Atlas screw, Lateral mass, Pedicle

## Abstract

**Background:**

Occipital neuralgia is one of the postoperative complications of C1 lateral mass screw insertion, which was deemed to be related with the C2 nerve root dysfunction.

**Case presentation:**

A 52-year-old female patient presented with gradually progressive numbness and weakness in her extremities for 6 months. X-ray and computed tomography (CT) scan revealed obvious anterior atlantoaxial dislocation (ADD), which was reducible on extensive view. Atlantoaxial pedicle screw fixation and bone graft was performed. Immediately after the operation, the neurological symptom significantly improved. The patient complained of restricted cervical rotation and suboccipital neuralgia which was exacerbated by rotation with an intensity of 7 on a visual analog scale (VAS) ranging from 0 to 10 at postoperative day 5. While a satisfactory reduction was detected in the postoperative CT, violation of the left atlantooccipital joint was observed in the left C1 screw. Nimesulide (daily dosage of 0.2 g) and bracing were recommended immediately. At the 2 month follow-up, both the neurological improvement and reduction were maintained. The VAS of suboccipital neuralgia is 3 and decreased to 1 at 6 months postoperative. Bony fusion of the left atlantooccipital joint was confirmed by CT scan at 6 months postoperative. The patient complained that the suboccipital neuralgia was tolerable without the assistance of braces or medications for pain. At the 18 month follow-up, only stiffness of head flexion and rotation was observed without suboccipital neuralgia.

**Conclusion:**

Suboccipital neuralgia after atlantooccipital joint violation of C1 pedicle screw placement most likely results from C1 nerve root irritation. As the corresponding dermatome differs from the distributing region and aggravated factor of C2 nerve root dysfunction, neuralgia due to C1 irritation was only localized at suboccipital region and exacerbated by rotation.

## Background

Occipital neuralgia (ON) is one of the postoperative complications of C1 lateral mass screw insertion which compromises the quality of life of patients and has been characterized by lancinating pain and dysesthesia in the occipital region. Compared to the current C1 lateral mass screw technique, C1 pedicle screw fixation which was first reported by Resnick and Benzel in 2002, is becoming the most popular technique for atlantoaxial fixation. The latter is performed with lower incidence of irritation to the C2 nerve root, a stronger pullout strength and less blood loss from the venous plexus [1–5]. In this case study, we will present a patient with severe suboccipital pain after atlantoaxial pedicle screw fixation, which was attributed to atlantooccipital joint violation of unilateral C1 pedicle screw insertion.

## Case presentation

**Fig. 1:**
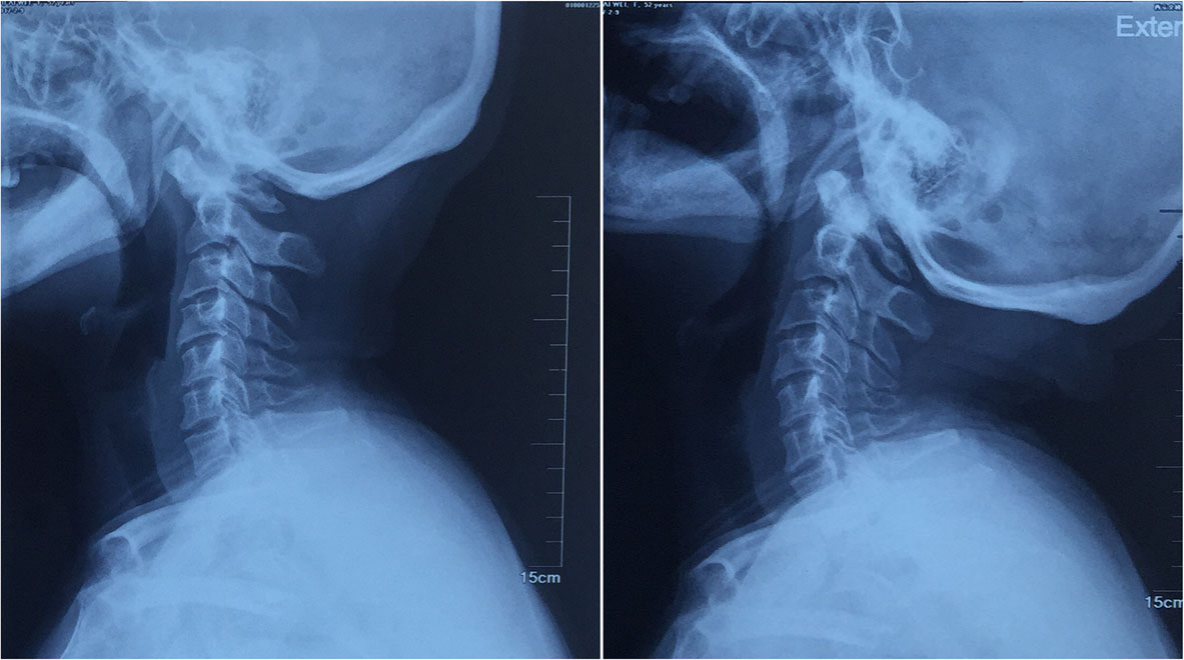
X-ray revealed obvious anterior atlantoaxial dislocation (left), which was reducible on extension view (right)

**Fig. 2:**
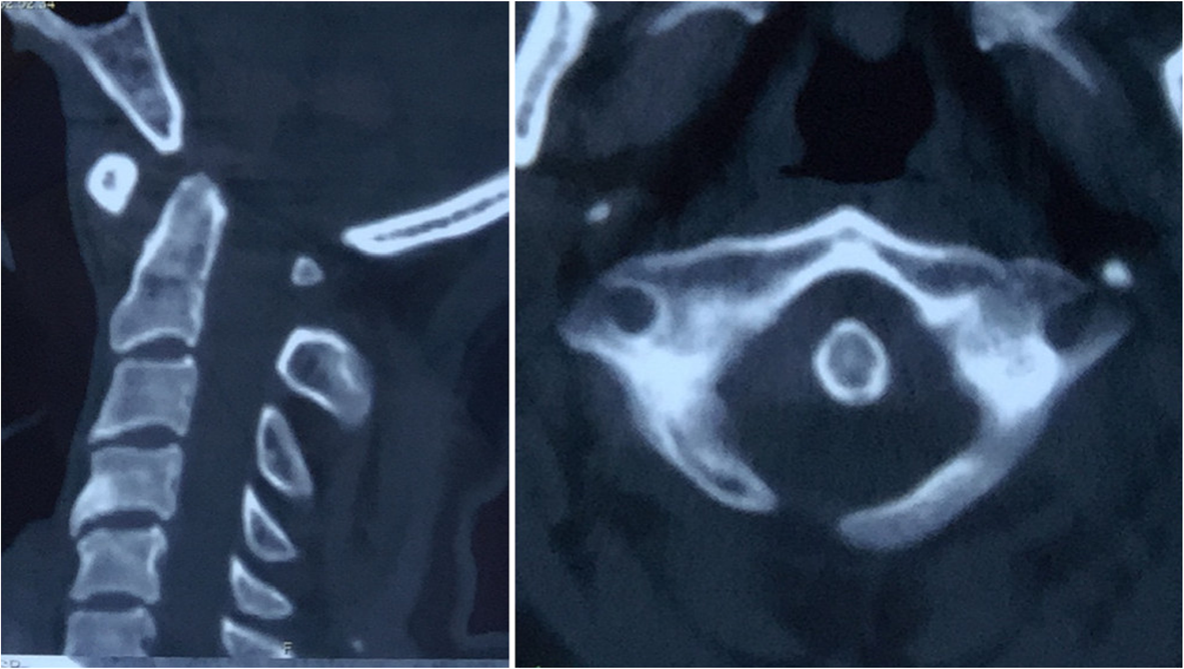
Computed tomography (CT) scan revealed obvious anterior atlantoaxial dislocation

**Fig. 3:**
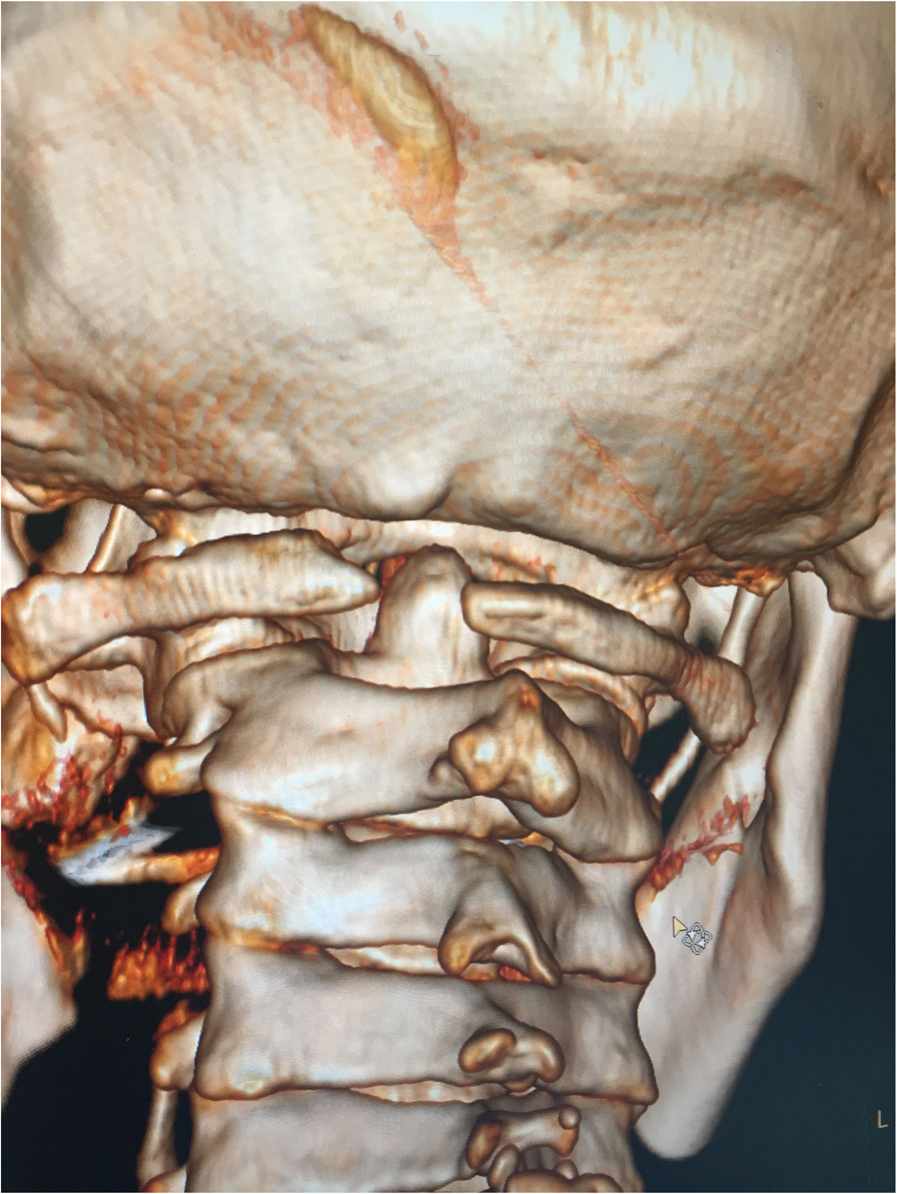
The left side of the posterior arch was revealed as the site of defect on CT 3-dimensional imaging

A 52-year-old female patient presented with gradually progressive numbness and weakness in her extremities for 6 months. No obvious history of injury was reported. Physical examination evaluated the weakness of the extremities (grades 4–5 strength), especially for the bilateral handgrip muscle (grades 3–5 strength). The deep tendon reflexes in all extremities were suspected to be positive. X-ray and computed tomography (CT) scan revealed obvious anterior atlantoaxial dislocation (ADD), which was reducible on extensive view (Figs. 1 and 2). The left side of the posterior arch was revealed as the site of defect on CT 3-dimensional imaging (Fig. 3).

During the surgery, the patient was placed in a prone position under general anesthesia, the neck was slightly extended with a Mayfield head holder. It was confirmed that the ADD was satisfactorily reduced under fluoroscopy. According to the C1–2 pedicle screw fixation technique reported by Resnick and Benzel [1], bilateral polyaxial screws were placed freehand into the C1 and C2 pedicles and connected by 2 separated titanium rods. Decortication was accomplished by a high-speed burr into the right side of C1 and C2 lamina, and an autologous bone graft was performed.

**Fig. 4:**
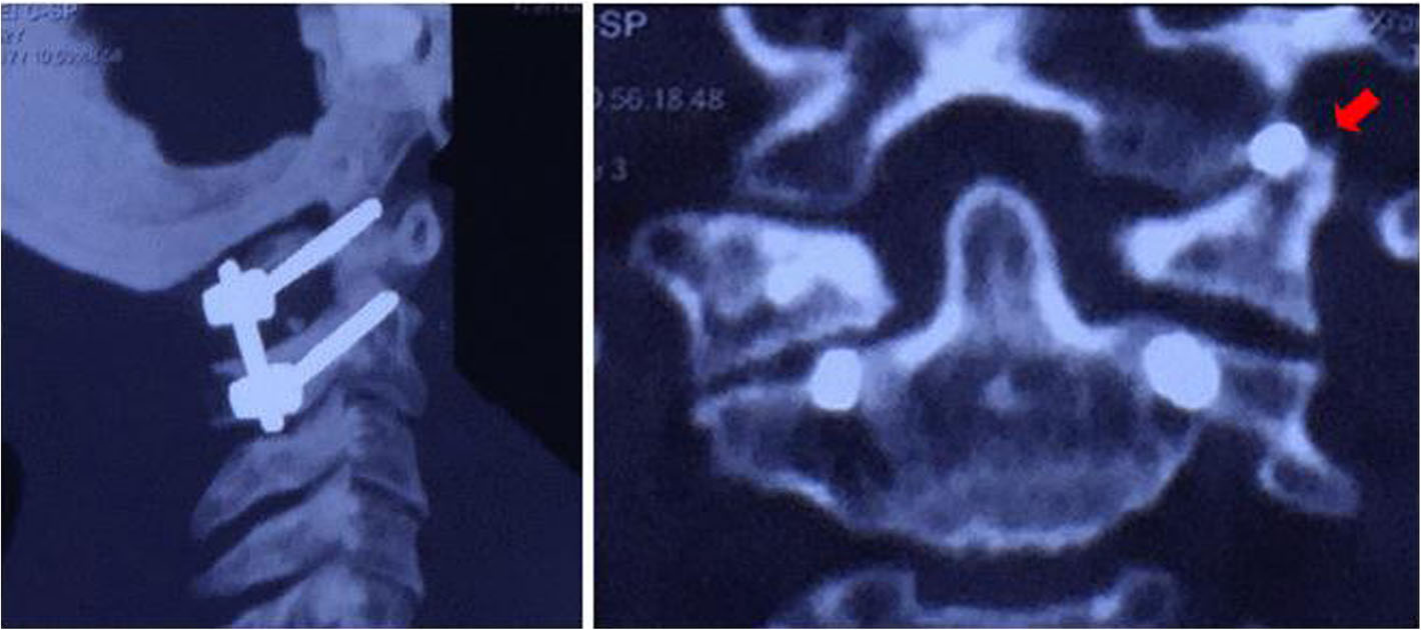
While a satisfactory reduction was detected in the postoperative CT, violation of the left atlantooccipital joint was observed in the left C1 screw (arrow)

**Fig. 5:**
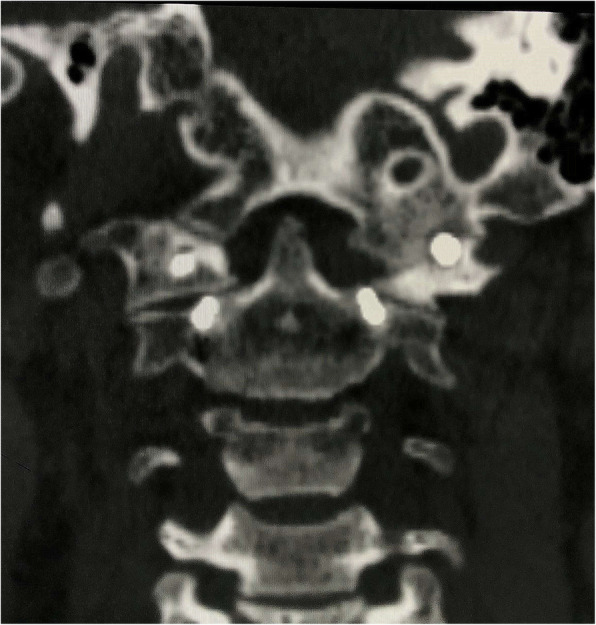
Bony fusion of the left atlantooccipital joint was confirmed by CT scan at 6 months postoperativ

Immediately after the operation, the neurological symptom significantly improved. The patient complained of restricted cervical rotation and suboccipital neuralgia which was exacerbated by rotation with an intensity of 7 on a visual analog scale (VAS) ranging from 0 to 10 at postoperative day 5. While a satisfactory reduction was detected in the postoperative CT, violation of the left atlantooccipital joint was observed in the left C1 screw (Fig. 4). Nimesulide (daily dosage of 0.2 g) and bracing were recommended immediately. At the 2 month follow-up, both the neurological improvement and reduction were maintained. The VAS of suboccipital neuralgia is 3 and decreased to 1 at 6 months postoperative. Bony fusion of the left atlantooccipital joint was confirmed by CT scan at 6 months postoperative (Fig. 5). The patient complained that the suboccipital neuralgia was tolerable without the assistance of braces or medications for pain. At the 18 month follow-up, only stiffness of head flexion and rotation was observed without suboccipital neuralgia.

## Discussion and conclusions

Screw violation of the facet capsule and/or the facet joint was widely explored in lumbar spine, which is believed to be associated with postoperative pain because of impingement and instability. In addition, the alterations of the normal biomechanics adjacent to the level of fixation may lead to earlier degeneration [6]. Recent studies on atlantoaxial fixation have elaborated that C1 lateral mass screw placement could induce C2 irritation which was deemed to be the determining factor in the pathogenesis of ON [2, 3, 5]. Huang, et al. speculates that the difference in height between the C2 ganglion and its corresponding foramen could determine whether the C2 ganglion is impinged; when the height left for the screw in the C1–2 foramen was < 4 mm, it could present a high risk of postoperative C2 nerve dysfunction [7]. Resnick and Benzel first reported the C1 pedicle screw fixation technique in 2002, which in essence inserted the C1 lateral mass screw via the posterior arch [1]. As the entry point was slightly lifted, compared to C1 lateral mass screw placement, it may provide lower incidence of ON, resulting from irritation to the C2 nerve root [4, 5]. The bilateral C2 ganglion was not sacrificed during the fixation process in our case, and the irritation to C2 ganglion was theoretically lower as the entry point was higher. Nevertheless, it could increase the chances of the screw violating the atlantooccipital joint for some cases with small lateral mass. Due to the change in intraoperative position change and individual differences, inserting the C1 pedicle screw by free hand might be hard in terms of staying on the recommended direction. The osseous overlap mainly concerns the craniocervical region as it could impair the quality of intraoperative fluoroscopy. The possibility of the C1 pedicle screw violating the atlantooccipital joint could be reduced during the surgical procedure.

As the occipital region is the corresponding dermatome of C1-C3, ON is not only related with C2 irritation, but also with C1 dysfunction. As the differences in the corresponding dermatome differed from the distributing region of C2 nerve root dysfunction; neuralgia due to C1 irritation was only localized at the suboccipital region. Screw violation of the atlantooccipital joint could induce impingement, which explained why suboccipital pain was exacerbated by rotation.

**Fig. 6:**
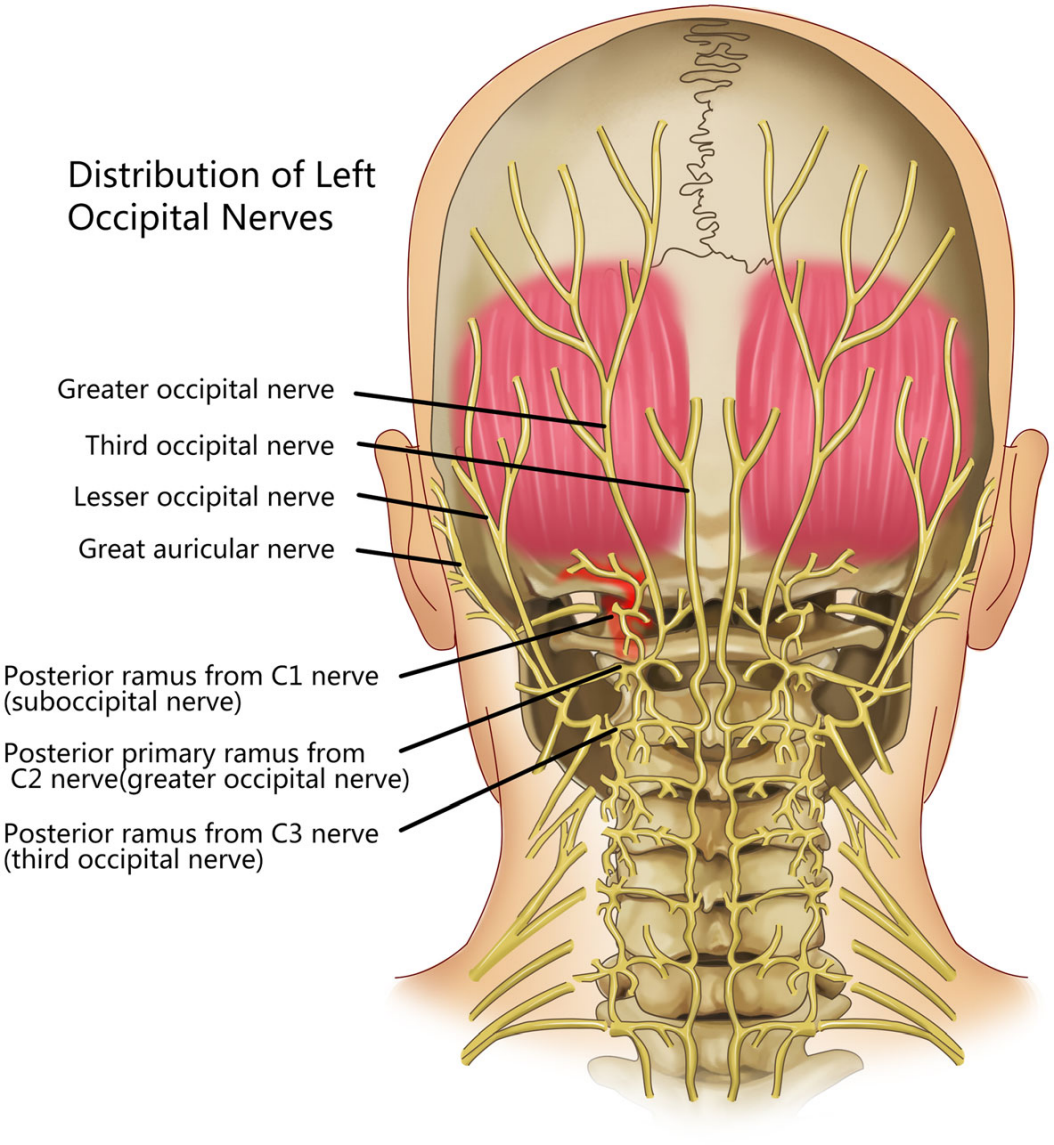
The suboccipital nerve is the dorsal primary ramus of the first cervical nerve, which was highlighted by red color

Additionally, ON, as a symptom, was not only reported in cases after atlantoaxial fixation, but also observed in certain cases of atlantoaxial osteoarthritis (AAOA) [8]. The pathogenesis of pain in ON has been postulated to include direct compression of the occipital nerves, as well as micromotion of the degenerated atlantoaxial facet joint itself, with pain transmission via the anterior ramus of the C2 [8, 9]. Atlantoaxial fusion could eliminate facet micromotion; thus expecting to remove one of the primary pain generators to some degree [10]. The suboccipital nerve is the dorsal primary ramus of the first cervical nerve (Fig. 6). The atlantooccipital joint has a large range of rotation in the cervical spine secondary to the atlantoaxial joint. Similar with facet violation in the lumbar spine, screw violation of the atlantooccipital joint could induce impingement and impair the joint surface, thus leading to traumatic arthritis. Additionally, atlantooccipital joint degeneration could also generate micromotion. Any inflammatory irritation or mechanical instability could generate neuralgia which could affect the surrounding neural structure - the posterior ramus of C1, the so called suboccipital nerve. The progression of osteoarthritis could lead to fibrosis or bony ankylosis. Similar as to the effectiveness of C1–2 fusion was for patients with intractable neck pain secondary to AAOA. The suboccipital neuralgia was finally resolved when bony fusion was formed at the left atlantooccipital joint.

ON is commonly idiopathic but may occur when the C1–3 nerve roots are statically or dynamically compressed after occipitocervical surgery. Suboccipital neuralgia after atlantooccipital joint violation of C1 pedicle screw placement most likely results from C1 nerve root irritation. As the corresponding dermatome differs from the distributing region and aggravated factor of C2 nerve root dysfunction, neuralgia due to C1 irritation was only localized at suboccipital region and exacerbated by rotation. In such cases, screw violation into the atlantooccipital joint after atlantoaxial pedicle screw fixation could be a neglected cause of suboccipital pain.

## Data Availability

All data are included in the section of Case Presentation and are available from the corresponding author on reasonable request.

## Abbreviations

AAOA: Atlantoaxial osteoarthritis
ADD: Anterior atlantoaxial dislocation
CT: Computed tomography
ON: Occipital neuralgia
VAS: Visual analog scale
